# Non-Invasive Intranasal Delivery of *pApoE2*: Effect of Multiple Dosing on the ApoE2 Expression in Mice Brain

**DOI:** 10.3390/ijms241613019

**Published:** 2023-08-21

**Authors:** Avinash Gothwal, Richard Nii Lante Lamptey, Riddhi Trivedi, Bivek Chaulagain, Jagdish Singh

**Affiliations:** Department of Pharmaceutical Sciences, School of Pharmacy, College of Health Professions, North Dakota State University, Fargo, ND 58105, USA; avinash.gothwal@ndus.edu (A.G.); richard.lamptey@ndsu.edu (R.N.L.L.); riddhi.trivedi@ndsu.edu (R.T.); bivek.chaulagain@ndsu.edu (B.C.)

**Keywords:** intranasal, multi-functionalized chitosan micelles, gene delivery, brain targeting, polyplex

## Abstract

Chitosan-based polymeric micelles are promising non-viral nanocarriers for safe and targeted gene delivery. Multi-functionalized chitosan polymeric micelles were prepared by grafting fatty acid, cell-penetrating peptide, and mannose on the chitosan backbone. The polymeric micelles were subjected to surface morphology and surface topography using scanning electron microscopy and atomic force microscopy, respectively. The hemotoxic profile of the prepared polymeric micelles was established against erythrocytes and was found to be <5% hemotoxic up to the concentration of 600 µg/mL. In vitro *ApoE2* expression in primary astrocytes and neurons was analyzed. Multi-functionalized polymeric micelles produced greater (*p* < 0.05) transfection in astrocytes and neurons in comparison to mono-functionalized micelles. Intranasal administration of polymeric micelles/*pApoE2* polyplex led to significantly higher (*p* < 0.05) in vivo *pApoE2* expression than chitosan and unfunctionalized polymeric micelles-treated mice groups. The outcomes of this study predict that the developed multi-functionalized polymeric micelles could be an effective and safe gene delivery platform to the brain through the intranasal route.

## 1. Introduction

Gene therapy is a promising therapeutic strategy for the prevention or treatment of diverse disease conditions by altering or knocking out defective genes [1,2]. However, gene therapy for Alzheimer’s disease (AD) is still a quandary and has proven to be an expensive failure in most clinical trials [3]. This situation has yielded stagnation to the introduction of potential therapies for AD management, and also motivation to scientists to investigate new disease-modifying effective gene therapy for AD. It has been accepted that, in late-onset AD (LOAD) cases, polymorphism in the apolipoprotein E gene (ApoE) is the major risk factor [4]. The influential role of ApoEs in AD progression is complex and multifactorial. Elevated expression of *ApoE4* increases the risk of AD by several folds [5]. Conversely, higher expression of *ApoE2* level decreases the risk of developing LOAD [6]. This suggests that *pApoE2* gene therapy could be a better therapeutic approach in AD management. However, naked gene delivery to the targeted organs is quite challenging due to the vulnerability of genes to nuclease degradation, poor cellular uptake, and transfection [7]. Moreover, the presence of the BBB in the brain also restricts the entry of exogenous molecules due to its restrictive and selective permeability nature [8,9]. On the other hand, the intranasal route of administration offers a non-invasive direct gateway to reach the brain through trigeminal and olfactory nerves by bypassing the BBB. The innervation of the upper nasal cavity with these nerves transports the drug molecules to the cerebrum and pons in the brain, where the drug molecules disperse in the whole brain aided by fluid movement [10]. Chitosan is a cationic polymer of natural origin comprised of β (1,4) linked D-glucosamine and N-acetyl-D-glucosamine units [11]. Chitosan-based polymeric micelles have shown promising gene delivery and transfection capabilities as a non-viral gene vector [1,2,12]. Chitosan-based vectors are bio-degradable, bio-compatible, and less immunogenic, and can be chemically modified to achieve specific delivery goals [11]. Additionally, protonation of primary amines under acidic conditions of chitosan is favorable to anionic pDNA interaction and retainment of cationic charge even after, which makes chitosan an ideal polymer for gene delivery [13].

Cell-penetrating peptides have been extensively investigated for brain-targeted delivery due to their specificity to receptors. Several studies have reported higher transfection by targeting ligand-modified chitosan-based nano-carriers [2,14,15,16,17]. Studies have shown the introduction of mannose (MAN) and penetratin (PEN) to nanocarriers led to improved uptake [2,18,19,20,21,22,23]. Insertion of fatty acid on CS results in amphiphilic cationic polymer, which has the ability to self-assemble in the aqueous environment [11]. The grafting of fatty acid and cell-penetrating peptides on chitosan can improve the targeting potential to deliver pDNA to the brain, resulting in higher transfection. Multi-functionalized chitosan polymeric micelles will improve brain transfection by effective translocation of pDNA through trigeminal and olfactory nerves. Unfortunately, limited research has been conducted so far using cell penetratin peptide grafted polymeric nano-micelles. 

This study focuses on multi-functionalized chitosan polymeric nano-micelle-based pApoE delivery to the brain through the intranasal route. Elevated ApoE expression has been documented in animals to attenuate AD progression [6]. The purpose of this study was to develop a safe and efficient pDNA (*pApoE2*) therapy for AD using multi-functionalized chitosan polymeric nano-micelles through the intra-nasal route. To achieve higher transfection in the brain, chitosan (CS) was modified with oleic acid (OA), penetratin, and mannose. Synthesized conjugates were characterized and evaluated for their toxicity and gene delivery potentials in in vitro and in vivo. 

## 2. Results

### 2.1. Scanning Electron Microscopy (SEM) and Atomic Force Microscopy (AFM)

**Figure 1:**
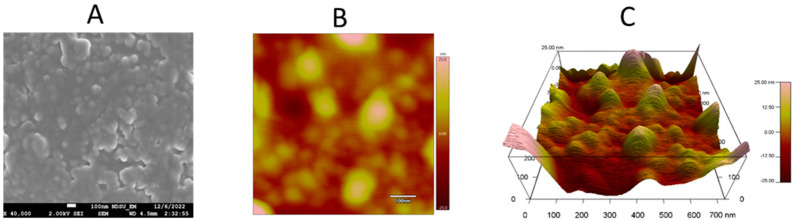
(**A**) Scanning electron microscopic (SEM) image of OA-g-CS-PEN-MAN polymeric micelles; (**B**,**C**) Atomic force microscopic (AFM) images of OA-g-CS-PEN-MAN polymeric micelles.

The morphology of the OA-g-CS-PEN-MAN polymeric micelles was evaluated using scanning electron microscopy. Figure 1 shows images of polymeric micelles. The SEM revealed that the polymeric micelles were spherical in shape and uniform in size distribution (Figure 1A).

Atomic force microscopy was used to visualize the surface topography of OA-g-CS-PEN-MAN polymeric micelles. The surface of the polymeric micelles was smooth, as can be observed in Figure 1B,C, the height of the sample was 25 nm.

### 2.2. Selected Area Electron Diffraction (SAED) and X-Rya Diffraction (X-RD)

**Figure 2:**
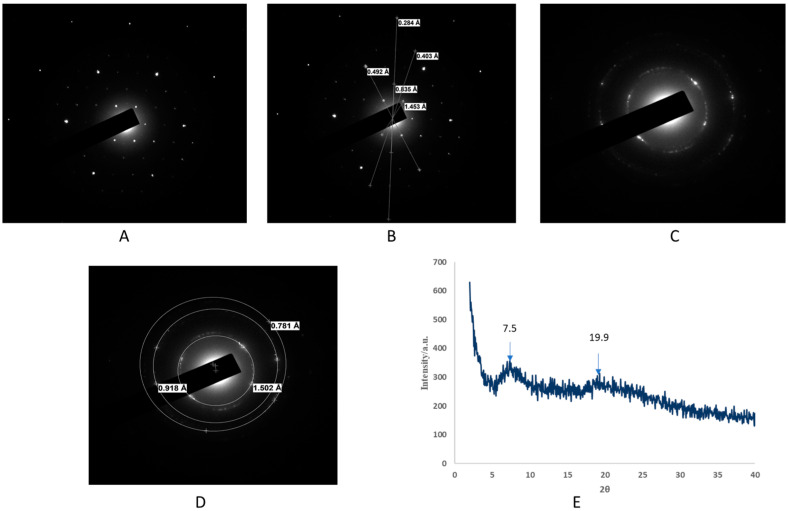
(**A**–**D**) Selected Area Electron Diffraction (SAED) patterns of OA-g-CS-PEN-MAN polymeric micelles; (**E**): X-ray diffraction (X-RD) pattern of OA-g-CS-PEN-MAN polymeric micelles.

The non-crystalline phase of the OA-g-CS-PEN-MAN polymeric micelles was investigated using selected area electron diffraction (SAED). Briefly, a parallel beam of electrons bombarded a thin layered sample using a transmission electron microscope which led to the illumination of the sample. Due to larger spacing between atoms, the electrons diffract and scatter at particular angles, which forms a diffraction pattern. The multiple scattering patterns of OA-g-CS-PEN-MAN were found to be non-crystalline/amorphous, as can be seen in Figure 2A–D. Further, it was confirmed with the irregular interplanar distance in ring SAED patterns. The multiple scattering and ring SAED patterns were disordered and suggest that the OA-g-CS-PEN-MAN conjugate is an amorphous solid. Further, X-RD pattern results confirmed the amorphous nature of the conjugates. Figure 2E shows two peaks in the X-RD plot at 7.4° and 18.9° due to amorphous structure. 

### 2.3. Hemolysis

**Figure 3:**
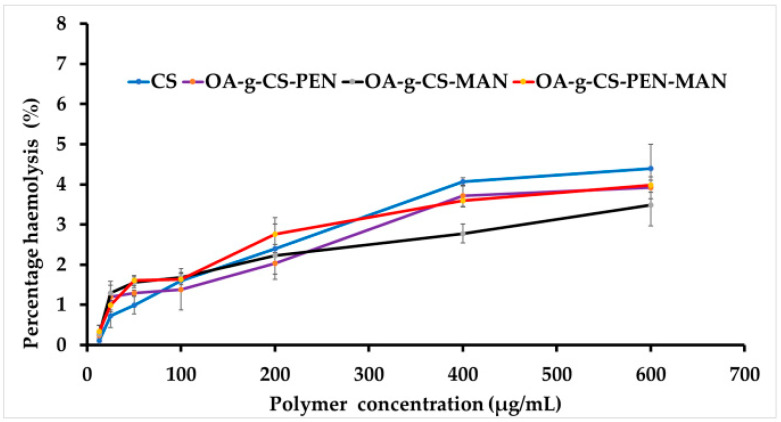
Represents hemolysis activity of CS, OA-g-CS, OA-g-CS-PEN, OA-g-CS-MAN, and OA-g-CS-PEN-MAN conjugates at different concentrations.

A non-specific interaction between cationic polymers and anionic membranes of erythrocytes may cause hemolysis and can affect the safety of the delivery systems adversely [11]. It also affects the targeting ability, half-life of the delivery system, and therapeutic efficacy [12]. The percent hemotoxicity of all the polymers was less than 5% at all concentrations (Figure 3). Generally, hemolysis of less than 10% is considered non-hemotoxic. Our polymers did not exert any hemotoxicity and were safe for further in vivo experiments.

### 2.4. In Vitro ApoE2 Transfection

**Figure 4:**
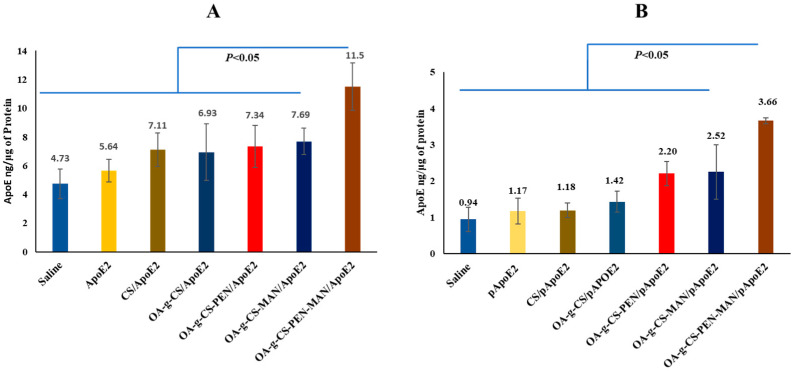
(**A**) Represents ApoE expression levels in primary astrocytes and; (**B**) in primary neurons after treatment with saline, *pApoE2*, CS/*pApoE2*, OA-g-CS/ *pApoE2*, OA-g-CS-PEN/ *pApoE2*, OA-g-CS-MAN/ *pApoE2*, OA-g-CS-PEN-MAN/*pApoE2* polyplexes. Data represented as mean ± SEM, n = 6. One-way ANOVA was used for statistical analysis.

An ideal non-viral vector should deliver the gene to the target site with safety and efficiency to induce elevated expression of the desired gene at the site. Primary astrocytes and neurons were used to evaluate the targeting potential of the OA-g-CS-PEN-MAN polymeric micelles. The OA-g-CS-PEN-MAN/*pApoE2* polyplex demonstrated significantly (*p* < 0.05) higher transfection of *pApoE2* in the primary astrocytes over naked *pApoE2*, approx. 1.9 times higher (Table 1, Figure 4A). Similarly, a significantly higher transfection was exerted by OA-g-CS-PEN-MAN/*pApoE2* polyplex over CS/*pApoE2*, OA-g-CS/*pApoE2*, OA-g-CS-PEN/*pApoE2*, and OA-g-CS-MAN/*pApoE2*. 

In neurons, significantly (*p* < 0.05) higher ApoE2 protein expression was observed in OA-g-CS-PEN-MAN/*pApoE2* polyplex treated cells than the other treatments. Precisely, *pApoE2* treated cells expressed 1.17 ng/µg of protein ApoE2 while OA-g-CS-PEN-MAN/*pApoE2* polyplex treated cells expressed 3.1 ng/µg of protein ApoE2 (Figure 4B). 

### 2.5. In Vivo pApoE2 Transfection

**Figure 5:**
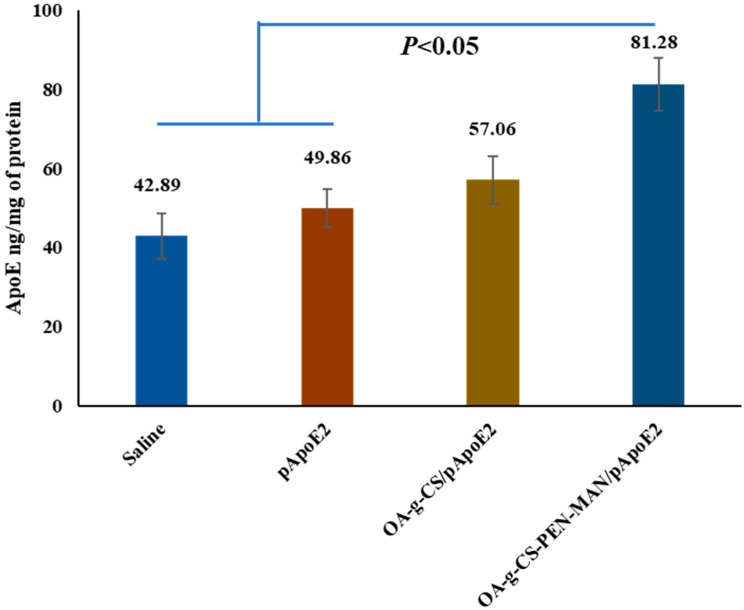
In vivo ApoE expressions in *C57BL6/J* mice after treatment saline, *pApoE2*, CS/*pApoE2*, OA-g-CS/*pApoE2*, OA-g-CS-PEN-MAN/*pApoE2* polyplexes. Data represented as mean ± SEM, n = 6. One-way ANOVA was used for statistical analysis.

To achieve the desired therapeutic effect in the brain, it is essential to transport and release the gene into the brain by the vector. Therefore, targeting and transfection potential of OA-g-CS-PEN-MAN/*pApoE2* polyplex was investigated in *C57BL6/J* mice following 7 days of intranasal administration. The animals were sacrificed on the seventh day of the last administration to harvest the organs to evaluate the ApoE2 expression levels in the brain using the ApoE2 ELISA kit. In vivo ApoE2 expression in the OA-g-CS-PEN-MAN/*pApoE2* treated animals was ≈1.63 times higher (*p* < 0.01) than the naked *pApoE2* treated animals. Furthermore, the ApoE2 expression levels in OA-g-CS/*pApoE2* polyplex and saline-treated animal groups were 57.06 ± 5.6 ng/mg, and 42.9 ± 5.7 ng/mg of protein, respectively (Figure 5). The naked *pApoE2* treated mice group exerted 49.86 ± 4.75 ng/mg of protein, non-significantly higher than the saline group.

## 3. Discussion

Chitosan has been extensively investigated for gene delivery due to higher gene packaging capacity and high possibilities of tailor-made modifications. Moreover, chitosan offers several advantages unlike other cationic polymers, such as less toxicity, less immunogenicity, higher cationic charge, and higher buffering capacity [24,25]. A higher positive charge facilitates the electrostatic interaction with negatively charged nucleic acids. Mounting evidence has suggested that high buffering capacity (pH 4.5–7.00) helps in escaping from endosomal or lysosomal degradation [26,27]. These properties make chitosan a polymer of interest for nucleic acid delivery.

Increasing evidence suggests that gene therapy has the potential to treat a wide range of ailments as a therapeutic agent [28,29,30,31]. The major challenge in gene therapy is the need for a vector to deliver the gene at the target site due to its vulnerability to enzymatic degradation. Mannose-modified polymeric nano-carriers are widely used for targeted gene or drug delivery to the brain [28,32]. This is due to the abundance of mannose receptor (GLUT-1) receptors on the BBB. Additionally, the GLUT-1 receptors are also present in neurons, so can be used for targeted gene delivery to the brain via the intranasal route [33]. 

The synthesized polymers were able to form polymeric micelles with a uniform size of <200 nm, uniformly distributed, and with cationic charge [34]. The cationic charge helps in higher encapsulation of the negatively charged gene [2,11]. The introduction of hydrophobic oleic acid on the CS backbone leads to an amphiphilic cationic polymer with self-assembling properties in an aqueous environment and forms micelles [12]. All the synthesized conjugates were able to form the micelles and protect the pDNA from enzymatic degradation [34]. 

Morphological characterization of the OA-g-CS-PEN-MAN polymeric micelles proved the spherical shape and uniform distribution. Further AFM results also confirmed the smoother surface and size. Spherical shape nanocarriers are more stable due to the least surface area per unit volume and interfacial energies [35]. Spherical nanocarriers exhibit higher cellular uptake [35,36]. The crystalline nature of chitosan depends on the degree of deacetylation [37]. Our synthesized OA-g-CS-PEN-MAN conjugate was amorphous as confirmed by scanning electron microscopy and X-RD. 

Optimum polymer-gene interaction is critical for binding and releasing the gene delivery at the cellular level. The OA-g-CS-PEN-MAN polymeric micelles are deprotonated at pH 6.5 and possess a cationic charge while due to lower pKa (~1) of the phosphate functional groups, pDNA is negatively charged [38]. This helps in the polyplex formation via electrostatic interaction. A stronger interaction will restrict the pDNA release from the polyplex while a weak interaction will release it quickly. Our previous ITC findings suggest that functionalized chitosan established a feasible interaction with pDNA [39]. The OA-g-CS-PEN-MAN polymeric micelles were able to protect the pDNA from enzymatic degradation [34]. The capability of interaction with pDNA and protection from DNase make OA-g-CS-PEN-MAN polymeric micelles a reliable drug delivery platform.

The safety of the nanocarriers in biological systems is crucial. Cationic-charged nanocarriers cause hemolysis due to a non-specific interaction between erythrocyte membrane and nanocarriers. Hemotoxicity limits the use of these nanocarriers in animal experiments. However, surface modification limits the RBC-nanocarrier direct interaction. Our finding revealed, less than 5% hemotoxicity by our synthesized polymers, which allowed us to use the OA-g-CS-PEN-MAN polymeric micelles for further use [40]. 

The main objective of this study was to enhance the expression of ApoE2 in neurons and astrocytes in the brain. Thus, we analyzed quantitative ApoE2 expression in the astrocytes and neurons ex vivo. Comparatively, higher ApoE2 expression was observed in primary astrocytes than in neurons. The underlying mechanism for transfecting the cells is receptor-ligand-mediated cellular internalization. The ApoE2 expression in astrocytes and neurons was considerably higher in the OA-g-CS-PEN-MAN/*pApoE2* polyplex treatment than in other formulations. The ApoE2 expression in pApoE2 treated astrocytes was 1.9 folds lower than the OA-g-CS-PEN-MAN/*pApoE2* polyplex treated astrocytes. The site specificity of the PEN and MAN-conjugated polymeric micelles was higher due to the abundance of the GLUT-1 receptor on astrocytes and neurons. This helps in achieving higher transfection than the unmodified polyplexes and naked *pApoE2*. As per the previous reports, ApoE2 protects brain cells including pericytes, endothelial cells, and oligodendrocytes [41,42,43]. 

Localized accumulation of functionalized nanocarrier to the brain is essential for the expression of the delivering gene. Based on in vitro results, the transfection potential of the OA-g-CS-PEN-MAN polymeric micelles was evaluated in *C57BL/6J* mice. Four treatment groups were selected based on our in vitro results, where saline and *pApoE2* were used as a control. The non-viral vector expresses the transgene for up to 6 weeks. Based on our previous work, we selected a 7-day treatment for the in vivo ApoE expression in *C57BL6/J* [2,34,44]. The mice group, treated with OA-g-CS-PEN-MAN/*pApoE2* was significantly (*p* < 0.05) higher than the saline, *pApoE2*, and OA-g-CS/*pApoE2* treated groups. Airway epithelial sensory neurons and trigeminal nerves offer direct access to the brain. Our findings also support the notion of the use of the intranasal route for the effective delivery of bio-actives to the brain for improved therapeutics.

## 4. Materials and Methods

### 4.1. Materials

Cytosin -β-D-arabinofuranoside, and RIPA buffer were purchased from Sigma-Aldrich (St. Louis, MO, USA). The Micro BCA protein assay kit was bought from Pierce Biotechnology Inc. (Rockford, IL, USA). Plasmid DNA encoding the green fluorescent protein (gWiz-GFP) and apolipoprotein E inducible (*pApoE2*) were acquired from Aldevron LLC (Fargo, ND, USA). Dulbecco’s modification of Eagle’s medium (DMEM) was purchased from Corning Incorporated (Corning, NY, USA). Dulbecco’s phosphate-buffered saline (DPBS) 1X and Penicillin streptomycin were purchased from VWR Chemicals, LLC, Sanborn, NY, USA. Neurobasal media was purchased from Gibco, Grand Island, NY, USA. The ApoE2 ELISA kit and Fetal Bovine Serum were purchased from Thermo-fisher Scientific, Frederic, MD, USA. All other reagents were of analytical grade and used without further modification.

### 4.2. Methods

#### 4.2.1. Synthesis and Characterization of OA-g-CS-PEN-MAN Conjugate

Synthesis and characterization of chitosan (CS, 50 kDa) conjugate, OA-g-CS, OA-g-CS-PEN, OA-g-CS-MAN, and OA-g-CS-PEN-MAN have been published in our previous report [34]. The conjugates were subjected to physical characterization, 1H-NMR, FT-IR, and critical micelles concentration. Further, the polymeric micelles were analyzed for N/P ratio optimization, gene loading, and pDNA protection potentials [34].

#### 4.2.2. Surface Electron Microscopy (SEM) and Selected Area Electron Diffraction (SAED)

The surface morphology of the developed micelles was analyzed using scanning electron microscopy (Cryo-SEM). Lyophilized OA-g-CS-PEN-MAN conjugate was used for the analysis. Briefly, the sample was mounted on conductive tape on a brass stub and gently tapped. The excess sample was blown out by implying airflow. The sample-mounted brass stub was visualized using JEOL JSM-6490LV (JEOL USA, Inc., Peabody, MA, USA) under a low vacuum with a laser beam (15 kV acceleration voltage) at 1500× magnification [45,46]. Selected area electron diffraction (SAED) was also performed to evaluate the non-crystalline phase of the synthesized OA-g-CS-PEN-MAN conjugate. The SAED is a technique to acquire a 2-D electron diffraction pattern to differentiate between crystalline and non-crystalline samples. 

#### 4.2.3. X-ray Diffraction

The OA-g-CS-PEN-MAN conjugate was subjected to X-ray diffraction phase identification using an X-ray diffractometer (Rigaku, Ultima IV X-ray Diffractometer, Woodlands, TX, USA). Briefly, the sample was placed on a glass cuvette and mounted in the instrument for analysis. The scanning wavelength and rate were 0.154 nm and 0.25°/min respectively. The analysis was done at 44 kV voltage and 50 mA current. The scanning scope of 2θ was 2° to 40° [46]. 

#### 4.2.4. Atomic Force Microscopy

The surface topography of the micelles was studied using a DI-300 AFM (Veeco, MN, USA) operating in tapping mode. Briefly, 3 μL of the polymeric micelles containing solution was placed onto mica plates (Muscovite Mica for AFM grade V-1; 15 × 15 × 0.15 mm^3^). The mica plates were kept for air drying followed by drying under a flow of nitrogen gas. A spring constant range of 1.2–6.4 N/m was used for the conical cantilever, and the resonant frequency range was kept at 47–90 kHz. A 10 nm tip curvature radius was employed [11].

#### 4.2.5. Hemocompatibility

To evaluate biocompatibility for in vivo applications, the interaction between the cationic polymeric micelles and anionic RBCs was evaluated. The blood was collected from the adult Sprague-Dawley rats and stored in polypropylene centrifuge tubes containing 0.5 M ethylenediaminetetraacetic acid disodium salt (EDTA-Na_2_) solution. RBCs were collected from the blood by centrifuging at 1500 rpm for 10 mins. The obtained pellet was washed thrice with 10 mM PBS and resuspended in PBS. Hemocompatibility of CS alone, as well as OA-g-CS-PEN, OA-g-CS-MAN, and OA-g-CS-PEN-MAN at different concentrations (12.5, 25, 50, 100, 200, 400, 600 µg/mL), was tested by incubating 100 µL RBC suspension with 900 µL of sample solution for 1 h at 37 °C. The supernatant was collected after centrifuging the samples for 10 min at 1500 rpm and analyzed using a spectrophotometer at 540 nm. PBS was used as negative and 1 % Triton X-100 was used as negative and positive controls. Percent hemolysis was calculated using the given formula [11].

$$\mathrm{Percent}\;\mathrm{hemolysis}=\frac{A_{Polymer}-A_{PBS}}{A_{1\%\;Triton\;X-100}-A_{PBS}}\times 100$$

*A_Polymer_* is the absorbance of the polymer incubated with RBCs, *A_PBS_* is the absorbance of the sample incubated with PBS, and *A_1% Triton X-100_* is the absorbance of the sample in 1% *v*/*v* Triton X-100 solution.

#### 4.2.6. In Vitro *pApoE2* Transfection

The quantitative in vitro *pApoE2* transfection was performed in 24 well plates (1 × 10^5^ cells/well) using primary astrocytes and neuronal cells. Cells were treated with saline, *pApoE2* pDNA(1 µg), CS/*pApoE2*, OA-g-CS/*pApoE2*, OA-g-CS-PEN/*pApoE2*, OA-g-CS-MAN/*pApoE2* and OA-g-CS-PEN-MAN/*pApoE2* polyplexes in DMEM basal medium. After 4 h of incubation, treatment media was replaced with complete media and incubated for 48 h. Quantitative ApoE2 analysis in cell lysate supernatant media was done using the ApoE2 ELISA kit (Thermo Fischer Scientific, Waltham, MA, USA). The total amount of protein was normalized with BCA analysis [47].

#### 4.2.7. In Vivo pApoE2 Transfection

Polyplex formulations containing pApoE2 (1µg *pApoE2*/g body weight) were administered intranasally in *C57BL6/J* mice for seven days. Briefly, the animals were segregated into five groups consisting of 6 (3 males and 3 females) in each. The first and second groups were treated with saline and naked pDNA, respectively. At the same time, the third, fourth, and fifth groups were treated with CS/*pApoE2*, OA-g-CS/*pApoE2*, and OA-g-CS-PEN-MAN/*pApoE2* polyplexes, respectively. At administration, the animals were restrained in a supine position under anesthesia and administered 5 µL formulation in each nostril alternatively using a micropipette. After the treatment animals were placed into their respective cages. The administered volume was 20 µL/animal. Animals were treated for 7 days and sacrificed on the seventh day after the last dosing to collect the organs [2]. Tissue was homogenized utilizing RIPA buffer containing phosphatase and protease inhibitors to restrict protein degradation. The homogenate was centrifuged at 4000 rpm for 15 min at 4 °C to remove the debris. The supernatant was collected and analyzed for ApoE2 protein using an ELISA kit. 

## 5. Conclusions

Multi-functionalized chitosan polymeric micelles were prepared and characterized for pApoE2 delivery to the brain through intranasal administration. The polymeric micelles were spherical in shape and uniformly distributed. Our findings show the non-hemotoxic nature of the polymeric micelles against erythrocytes and higher transfection potential in astrocytes and neurons. Further, intranasal administration of OA-g-CS-PEN-MAN/*pApoE2* polyplex exhibited significantly higher expression of ApoE2 in the brain compared to non-functionalized polymeric micelles. This research reveals, functionalized chitosan polymeric micelles are potential nano-carrier platforms for targeted gene delivery to the brain through intranasal administration. 

## Figures and Tables

**Table 1:** ApoE expression in primary astrocytes, neurons, and *C57BL6/J* mice.

Treatment	ApoE Protein Expression (Mean ± SEM)		
	Astrocytes (ng/μg Protein)	Neurons (ng/μg Protein)	*C57BL6/J* Mice (ng/mg of Protein)
Saline	4.73 ± 1.03	0.94 ± 0.33	42.89 ± 5.76
*pApoE2*	5.64 ± 0.80	1.17 ± 0.35	49.86 ± 4.75
CS/*pApoE2*	7.11 ± 1.15	1.18 ± 0.20	-
OA-g-CS/*pApoE2*	6.93 ± 1.96	1.42 ± 0.29	61.76 ± 6.78
OA-g-CS-PEN/*pApoE2*	7.34 ± 1.47	2.20 ± 0.33	-
OA-g-CS-MAN/*pApoE2*	7.69 ± 0.93	2.25 ± 0.75	-
OA-g-CS-PEN-MAN/*pApoE2*	11.5 ± 1.64	3.66 ± 0.08	81.28 ± 6.71
